# Robust optimization of VMAT for lung cancer: Dosimetric implications of motion compensation techniques

**DOI:** 10.1002/acm2.12142

**Published:** 2017-08-08

**Authors:** Ben R. Archibald‐Heeren, Mikel V. Byrne, Yunfei Hu, Meng Cai, Yang Wang

**Affiliations:** ^1^ Radiation Oncology Centre Sydney Adventist Hospital Sydney NSW Australia; ^2^ Centre for Medical Radiation Physics University of Wollongong Wollongong NSW Australia

**Keywords:** inverse planning, lung, motion management, optimization, robust, VMAT

## Abstract

In inverse planning of lung radiotherapy, techniques are required to ensure dose coverage of target disease in the presence of tumor motion as a result of respiration. A range of published techniques for mitigating motion effects were compared for dose stability across 5 breath cycles of ±2 cm. Techniques included planning target volume (PTV) expansions, internal target volumes with (OITV) and without tissue override (ITV), average dataset scans (ADS), and mini‐max robust optimization. Volumetric arc therapy plans were created on a thorax phantom and verified with chamber and film measurements. Dose stability was compared by DVH analysis in calculations across all geometries. The lung override technique resulted in a substantial lack of dose coverage (−10%) to the tumor in the presence of large motion. PTV, ITV and ADS techniques resulted in substantial (up to 25%) maximum dose increases where solid tissue travelled into low density optimized regions. The results highlight the need for care in optimization of highly heterogeneous where density variations may occur with motion. Robust optimization was shown to provide greater stability in both maximum (<3%) and minimum dose variations (<2%) over all other techniques.

## 1 INTRODUCTION

Lung Cancer is the leading cause of cancer death in Australia^1^ whilst the American Cancer Society records 5‐year survival of lung tumor cases at 17%.^2^ Surgery morbidity and a need for multimodality treatments results in over half of all lung cancer patients receiving radiotherapy as some part of their clinical treatment.^2^ Volumetric Modulated Arc Radiotherapy (VMAT) and Intensity Modulated Radiotherapy (IMRT) have been shown to provide improvements in radiotherapy plan dose distribution over 3‐Dimensional Conformal Radiotherapy Techniques (3DCRT).^3^, ^4^

As a result of patient breathing, large variations in primary tumor position are often seen during radiotherapy of lung disease. An enormous amount of work has investigated the variations in organs and tumors with breathing. The AAPM Task Group 76^5^ presented the following summation on review of the literature; “The amount a lung tumor moves during breathing varies widely…There are no general patterns of respiratory behavior that can be assumed for a particular patient prior to observation and treatment”. Many of the reviewed studies^6^, ^7^, ^8^, ^9^, ^10^, ^11^, ^12^, ^13^ had focused on quantifying the magnitude of such tumor motion, showing variations as great as 34 mm, 22 mm, and 12 mm in the cranio‐caudal, anterior‐posterior, and lateral directions respectively, in some patients.^14^ Traditional planning methods provide suitable coverage of mobile Gross Tumour Volumes (GTV) by creation of Internal Target Volumes (ITV) which encompasses the GTV through its respiratory motion. Typically, a further expansion is made to account for geometric set‐up uncertainty of the patient, to create a Planning Target Volume (PTV) for which dose coverage metrics are assessed.

Recent improvements in the technology of 4‐Dimensional Computed Tomography (4DCT) image binning,^15^, ^16^, ^17^, ^18^ respiratory motion monitoring,^18^, ^19^, ^20^, ^21^, ^22^, ^23^ functional imaging correlation,^24^, ^25^, ^26^ and faster imaging techniques have resulted in several delivery methodologies to decrease the impact of lung motion. Another approach to reducing the impact is to minimize the motion itself by incorporating compression belts^27^ to restrict diaphragm contraction and expansion, implementing breath hold techniques^23^, ^28^, ^29^, ^30^ or gating the treatment by restricting delivery to particular components of the breathing cycle.^14^, ^20^, ^25^, ^31^, ^32^ Recent works from several research groups have also investigated the tracking of tumor by dynamic correction of MLC positions.^33^, ^34^, ^35^, ^36^, ^37^ In the majority of these solutions the objective is to minimize the ITV volume, and thus the PTV volume. Whilst some of the systems, as individual or combined solutions, are showing promising results in reduction in irradiated volumes and healthy tissue doses,^14^, ^23^, ^28^, ^38^ they provide a solution to only one half of the motion induced problem.

Calculation and delivery of modulated distributions on moving targets are subject to three well documented uncertainties between planning and delivery; blurring, interplay, and dose deformation.

McCarter and Beckham^39^ demonstrated large delivery variations in high dose gradient regions in the presence of tumor motion as a result of the blurring effect. Several authors^40^, ^41^ have shown that extreme dose variations of up to 100% in IMRT field delivery due to the interplay effect are theoretically possible. Subsequent statistical analysis by Bortfeld^42^ found no significant difference over long course treatments (>10 fractions) however, comparisons were made against an introduced formalism rather than a CT dose distribution (of which variations of up to 20% were noted). A study by Englesman et al.^43^ focused on dose deformation showed small variations (<5%) in dose distributions with tumor motion and a broadening of the high dose distribution along the axis of travel with 3D conformal planning.

Optimization is the computerization of mathematical problem solving. In the realm of radiotherapy planning the specific problem is the maximization of dose to a target volume whilst minimizing the dose to the surrounding tissue. By this definition a large component of the optimization process is ensuring a minimum dose to the voxels encompassed by a defined target volume.

A fourth potential issue in mobile lung disease is the impact of dose optimization to lung tissue and bronchial airways in the presence of tumor motion, where the objective function is required to ensure target dose coverage to large PTVs that include a volume of lung or air with a density significantly less than the GTV tissue.

Previous literature^44^, ^45^ has shown the adverse dosimetric implications of optimizing to and outside of surface contours, where the lack of electron density results in high photon fluence to achieve equivalent doses. In such cases when the patient tissue traverses into the region containing air during treatment, the high intensity fluence results in a sharp increase in primary interactions, liberated secondary electron generation, and resultant dose deposition.^46^, ^47^

In the situation of internal lung tissue the effect is less studied. The electron densities among air, lung, and muscle tissue is similar^48^ suggesting the effect is predominantly the result of physical density. It thus follows that an equivalent but reduced effect may be observed in lung/disease boundaries. In lung patients the result of this effect is complicated by a couple of further considerations;

1. Incident beams on lung tumors will undergo primary attenuation and build‐up of secondary electrons by superficial tissue, such as the muscles of the chest wall. These secondary electrons are of an order of magnitude less for surface optimization.
2. The movement is not limited to the target itself but can also include the surrounding tissue with no necessary correlation in direction or magnitude of motion.

Robust optimization is a recent introduction into the world of radiotherapy planning made possible by the increased parallel computational power of Graphic Processer Units (GPUs) along with more efficient threaded allocation of dose computations. Robust optimization allows a plan to be optimized such that it meets planning criteria in not only the planning geometry, but also in given patient and disease position variations.^45^ The commercial system used in this paper is Raystation v5.0.1 (Raysearch, Sweden).

**Figure 1:**
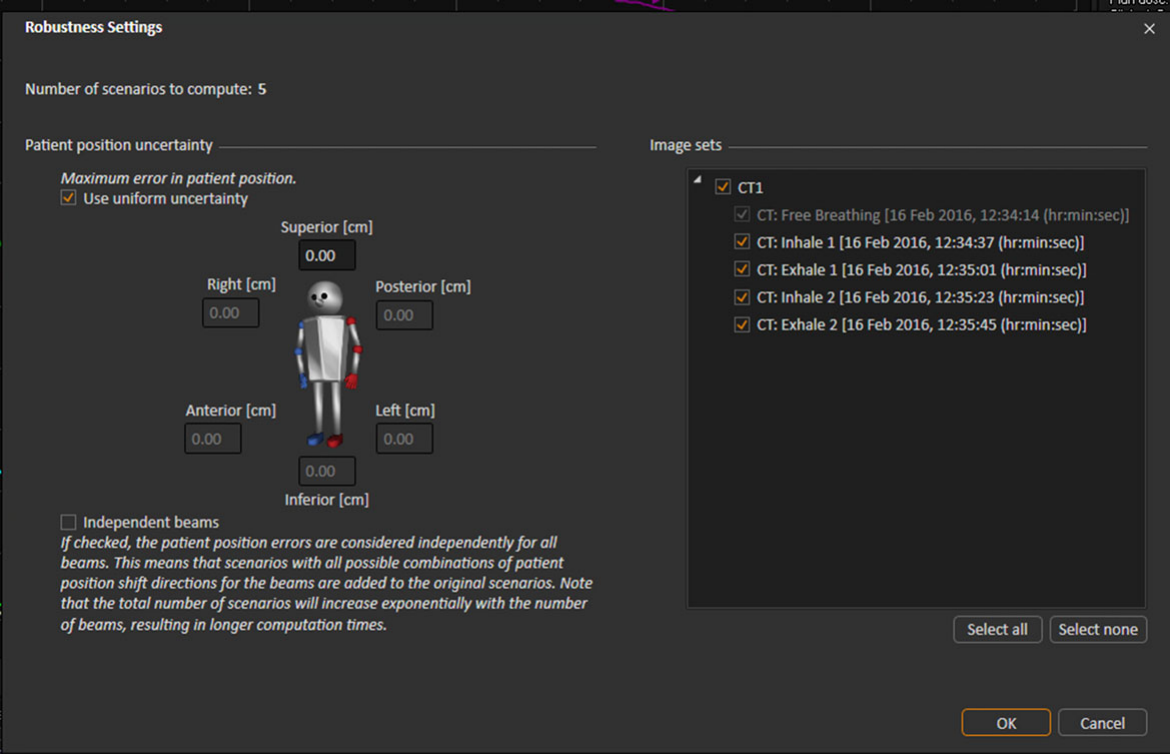
Robust optimization incorporation in Raystation using positional uncertainty (left) and multiple image sets (right).

Raystation ensures robust planning doses by the incorporation of min‐max optimization whereby the geometric uncertainties of the plan are incorporated in the problem function. The formalism includes no dependence on a probability distribution of the potential geometric uncertainty as per Bortfeld et al.,^49^ Chu et al.,^50^ Chan et al.,^51^ and Olafsson and Wright,^52^ but rather minimizes the objective function of the worst preforming geometry within the included distribution. This ensures a minimum level of plan quality, but results in a dependence on limitations of uncertainty and the potential for the system to over optimize low probability scenarios at the cost of plan quality of higher probability scenarios. In a paper by Fredriksson,^53^ in which the formalism was introduced, the method was shown to provide robust plans with increased lung sparing over PTV expansions for intensity modulated proton therapy, whilst work by Byrne et al^45^ has demonstrated its potential in IMRT planning. The implementation of min‐max optimization by the vendor is provided in two options; specified 3‐dimensional offsets set by the user or planning over a range of patient scans (Fig. 1).

The aim of this work is to establish the accuracy of the Raystation collapsed cone convolution algorithm in calculations across multiple datasets and to utilize this methodology to analyse the suitability of various optimization schemes in ensuring accurate and uniform dose to moving targets through breathing cycles in lung tissue.

## 2 METHOD

### 2.A Datasets

**Figure 2:**
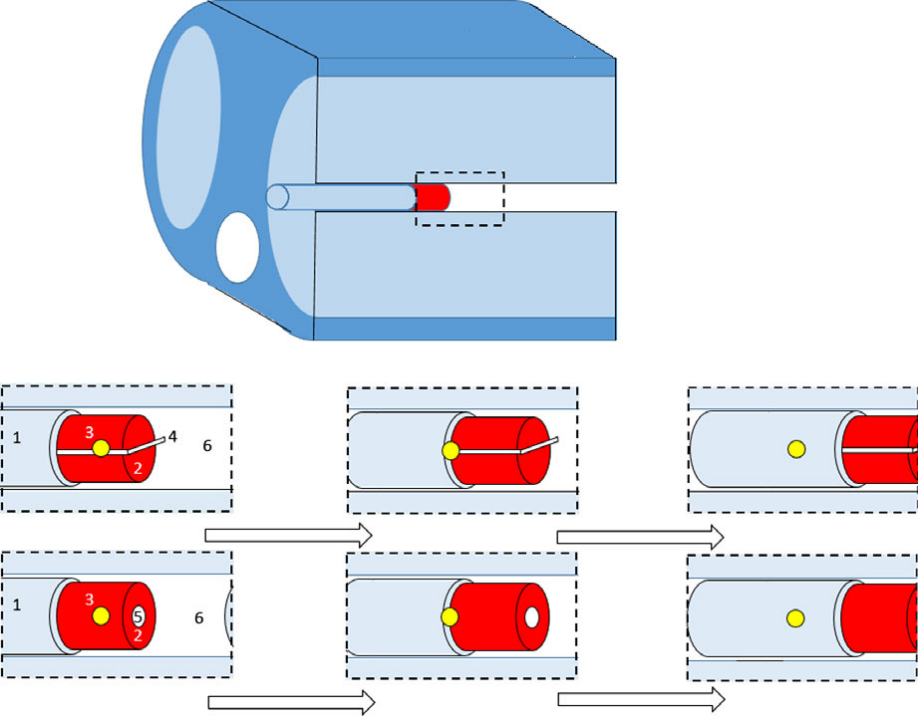
Shifts of GTV insert across scans. 1: Lung Tissue Insert, 2: GTV Wax Insert, 3: Isocentre, 4: Gafchromic Film, 5: Chamber insert, 6: Air Cavity.

Planning CT scans were taken with the CIRS thoracic phantom (CIRS Inc., Virginia, USA) with a set of custom made wax (average density 0.95 g/cm^3^) inserts. All scans were performed on an AS Definition CT scanner (Siemens Healthcare, Erlangen) at a maximum tube energy of 120 kVp, 216 mAs at 2 mm slices. Two separate wax inserts were used. The first wax insert was created with a chamber plug for an Exradin A1SL chamber (Standard Imaging, Wisconsin, USA), whilst the second insert included a Gafchromic film holder in the sagittal plane. The phantom was scanned in a breath cycle acquisition of five for each of the two chamber inserts, resulting in a total of 10 scans. It should be noted that the acquisitions were not true 4D binning, and had no time correlation. To emulate binned breathing the scans were taken with the phantom in an identical location but with the wax inserts manually translated in 1 cm increments from 2 cm inferior of the planning scan (0 cm offset) to 2 cm superior to the planning scan. For clarity the individual scans will be referred to by their offsets and collectively group referred to as the breathing cycle. In each of the CT scans of the film inserts, a dummy film was included to ensure the planning density matched that used for film exposures. This particular study is interested in the effect of optimizing to lung. To assess the extent of the effect the phantom insert region above the inserts was left as air, as demonstrated in Fig. 2.

For both the chamber and film cases the scans included the central planning position and two superior and inferior offset scans of 1 cm and 2 cm. Visual examples of the translations are shown in Fig. 2.

For each of the 4DCT sets an “average” scan was created in RayStation whereby the Hounsfield units of all five scans are summated and averaged on to a 6^th^ dataset. This was included to allow for the assessment of the potential use of this simpler and faster technique to account for varying geometry densities.

**Figure 3:**
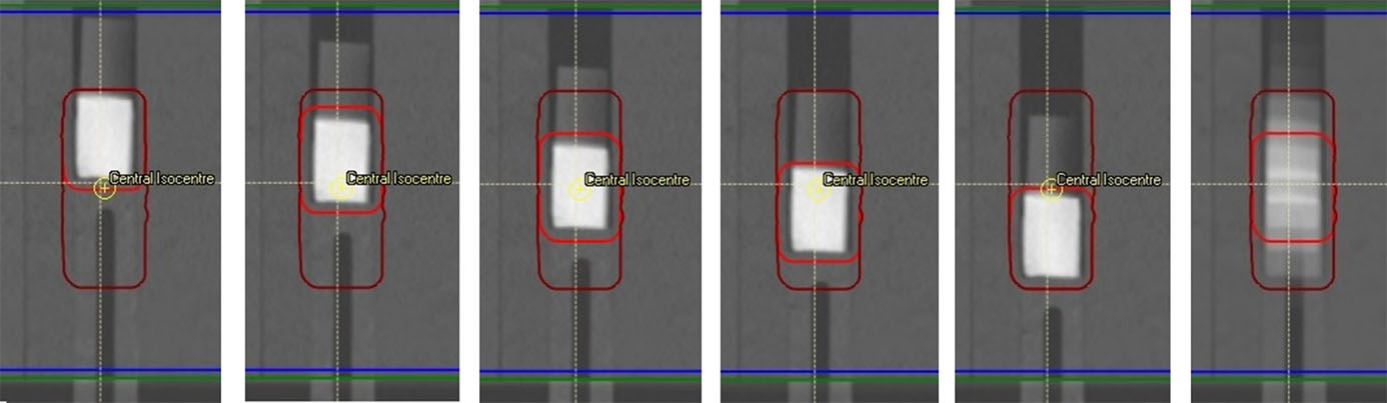
Travel of GTV superior to inferior (left to right) across all five scans, and the average compiled dataset (far right). Note the full travel of the GTV (red) is encompassed by ITV (maroon) geometry.

An example of each of the datasets are shown in Fig. 3 along with an example of the average dataset at the end. The sagittal orientation is shown for clarity of the average effect.

### 2.B Breathing cycles and plan parameters

All breathing cycle scans were imported into the Raystation system as 4DCT groups. Geometric contours representing the heart, left and right lungs, spinal cord and ribs were propagated across all datasets. The target volume was defined as GTV in each dataset individually, and an ITV was created as a summation of all GTVs registered back to the primary central dataset. A uniform expansion of 0.5 cm was applied to the GTV/ITV to create a PTV in each case. The margin was selected as per clinical protocol to account for imaging set‐up tolerances. All tumor motion margin was assumed to be included in the robust method incorporated.

To promote conformal dose distributions and to correlate with typical planning convention a ring geometry was created around the target ITV volume. An example of the target geometries is shown in Fig. 3, inclusive of a demonstration of the average 4DCT scan.

A set of plans were created for both the chamber dataset and film dataset, and are individually outlined below;

1. Plan ITV: Optimization was performed to ensure minimum dose to the full extent of the GTV travel. An ITV was created by summation of the GTV across all scans and a 5 mm PTV expansion applied in all orthogonal planes, inclusive of the air volume superiorly and lung volume inferiorly.
2. Plan GTV: Plan was optimized to the GTV in the central axis with a PTV expansion of 5 mm. It is expected that this will result in minimum coverage failures throughout the breathing cycle. This further highlights effects of edge of border effects at lung/tissue boundaries and expected dosimetry in a reduced phase treatment, such as gating.
3. Plan average: Plan was optimized to the ITV + 5 mm on an average dataset where the travel of the inserts resulted in a lower density at the central GTV position, but a spread medium density along the full length of travel. The volume was mapped to the average dataset by static registration to ensure comparable planning volumes. This technique has been proposed previously in the literature.^54^, ^55^ It should be noted that the average dataset approach does not consider the respective time component of each phase, but purely averages across all five scans. Thus, an area that is air in four of five scans and tissue of density 1.0 g/cm^3^ in the 5^th^ will result in an average CT density of the voxel of 0.2 g/cm^3^.
4. Plan robust all: Optimization was performed across all five datasets with GTV geometry travel. Only the maximum dose to lung, minimum PTV coverage and maximum PTV dose were included as robust objectives. All other objectives were only optimized on the central travel dataset.
5. Plan robust ext: Optimization was performed as per Robust All with the exception that only the central, the 2 cm superior, and the 2 cm inferior geometries were included in robust objectives. The intention of this test was to determine if the exclusion of intermediate breathing cycles would improve optimization speed without a reduction in the plan quality.
6. Plan robust values: Robust optimization was performed with user‐defined geometry offsets in the superior and inferior direction rather than across several scans. Robust optimization objectives were maintained as per the Robust All plan. This methodology offers the assessment of the potential of robust planning in the absence of multiple CT datasets rather than the conventional use of a uniform PTV expansion.
7. Plan lung override. Plans were optimized as per the methodology by Wiant et al^46^ where by the ITV excluding the GTV on the free breathing scan was overridden to an intermediate density. This methodology was shown to provide significant improvements over ITV optimization with the analytical anisotropic algorithm of the Eclipse planning system (Varian Oncology Systems, CA, USA). For this work a density override was set to 0.6 g/cm^−3^ to correlate with a midpoint density between solid tissue and the surrounding 0.2 g/cm^3^ lung.

Unless explicitly mentioned above, all plans were performed on the central planning scan. Each scan relied on a dual arc VMAT delivery of two 360°deliveries with the isocenter set to the centre of the GTV in the primary scan. To prevent excessive modulation, the leaf travel was limited to 0.5 cm/degree and the control point spacing set to 4° as per clinical practice. Plans were planned for a Varian iX iClinac with millennium MLC. Dose calculation was performed on a 3 mm dose grid.

A further set of square 10 × 10 Ant‐Post beams was added as a standard reference‐conditioned field to verify the accuracy of reference dose.

### 2.C Optimization

Prior to planning a set of clinical goals were set for the acceptance of plans. The goals were set arbitrarily to push the optimization system and create difficult but achievable modulated plans. The evaluation of clinical goals was performed solely on the planning (offset = 0 cm) geometry. These were loosely guided by common goals for typical dose and fractionation levels at the centre.

**Figure 4:**
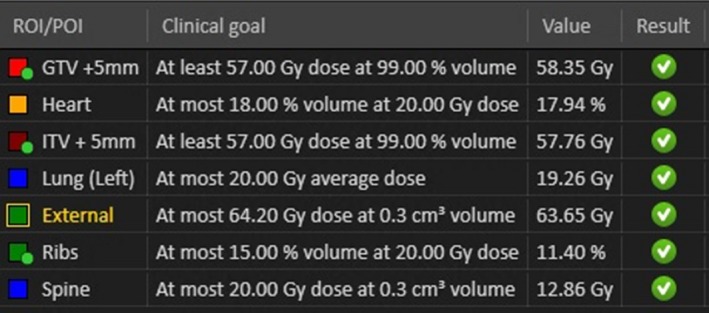
Plan clinical goals for optimization.

A list of the applied clinical goals is provided in Fig. 4.

For robust planning the motion of the ITV becomes redundant as the travel of the GTV is encompassed within the optimization system rather than a geometry expansion. For this reason, the ITV coverage was not optimized for robust plans.

Plans were accepted when they met the criteria set out in Fig. 4. In some scenarios these goals were exceeded. As plan quality was not a metric in this study, once clinical goals were satisfied the optimization ceased. As a result, the variation between final plan quality among all plans was negligible.

A concerted attempt was made to meet all clinical goals in each plan. In situations where goals could not be met, the plan was optimized such that the max dose control was the least critical. All but one plan of 14 (2 × 7 optimization techniques) met all clinical goals which exceeded the max constraint by <0.05% of TD.

For each case the final calculated dose, DVH curves, and dose statistics were recorded.

### 2.D Calculation on breath cycle

Each of the completed plans was recalculated on each phase of the 4DCT datasets. In Raystation, for CT datasets with identical UID and frame of reference, the plan isocenter is intrinsically correlated between datasets by the common DICOM co‐ordinates, providing consistency in set‐up with the exception of the moving lung insert. Comparisons were then made between DVH curves, organ statistics, and 2D dose distributions in each geometry. As the images were not taken as 4D binned sets calculation accuracy was reviewed on a per set basis independent of phase weightings.

### 2.E Measurements

Each of the created plans were exported to Mosaiq 2.4 (IMPAC, CA, USA). Plans were imported as per standard clinical practice and delivered to the CIRS phantom in the corresponding breath cycle phantom positions.

#### 2.E.1 Chamber measurements

**Figure 5:**
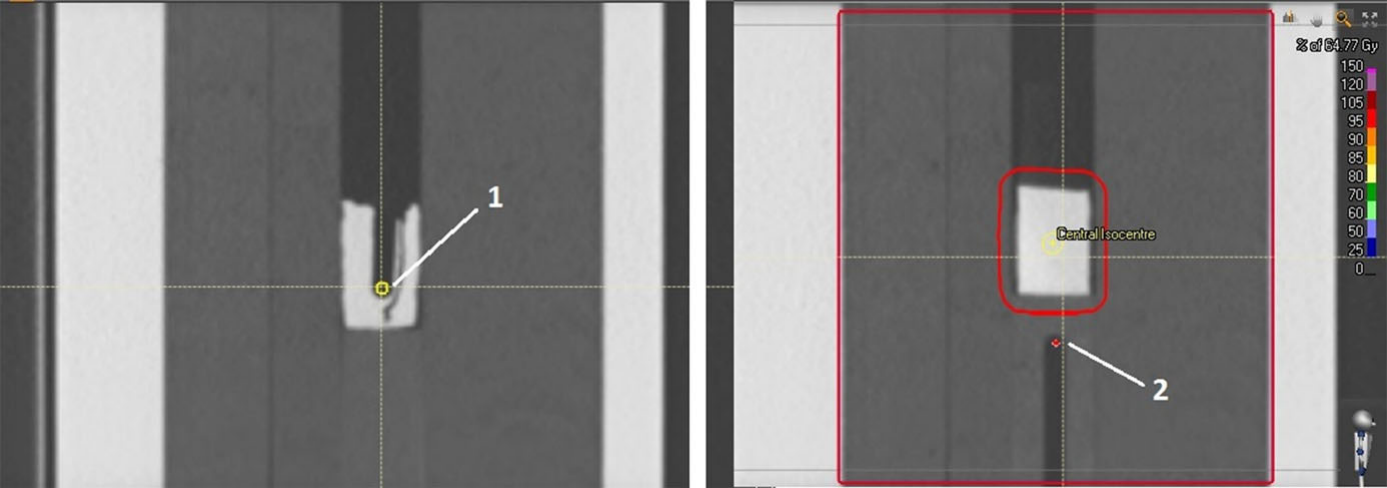
Position of chamber measurements for (a) GTV and (b) lung measurements.

For each plan two sets of chamber measurements were taken; one at the central tumor and one at the inferior lung. Chamber measurements at lung position were taken simultaneously with film measurements at a distance 1.5 cm inferior to the film insert as shown in Fig. 5 in the film plans. Central tumor measurements were taken with the A1SL chamber insert described above in separately optimized plans at the planned and offset positions as per film measurements. Dosimetry measurements were taken with Exradin A1SL (Standard Imaging, WI, USA) and PTW TN31010 Semiflex chambers with active volumes of 0.057 cm^3^ and 0.125 cm^3^ respectively. A PTW Webline electrometer (PTW, Freiburg, Germany), was utilized for charge collection and all corrections were applied as per the formalism of IAEA TRS 398 v12^56^ to convert the chamber reading into dose. All measurements were repeated twice. Before each measurement the chamber position was verified with on‐line Cone beam images to the original scans in RayStation.

Calculated chamber doses were determined by average dose to accurately modeled active volumes of the chamber geometries. Uncertainty for comparison was limited to positional errors of volume placement. As each delivery was imaged prior with CBCT positional accuracy of ±1 mm, errors were determined on a worst case 2 mm geometric set‐up error. Taking variations in average dose by shifting the active volume 2 mm in all planes across all plans resulted in an uncertainty of planned chamber dose of ±1.5% as a result of small positional errors. Measurements in low density material with Ion chambers are known to suffer from perturbation effects^57^ from the replacement of lung tissue and the nonwater equivalence the chamber walls, stem, and central electrode. As no current 6MV perturbation factors are published for the A1DSL chamber, all lung measurements were taken with the PTW Semiflex chamber. Work by Araki^58^ modelling the chamber with Monte Carlo calculations showed perturbation correction factors as much as −3% for 3 × 3 fields and −1% for 5 × 5 fields in 6MV beams. The predominant size of beams used in this work was approximately 5 × 3 cm and thus −3% represents a worst case scenario in the subsequent measurements. These perturbation factors were not applied to the final measurements, but were included within the total chamber uncertainty of ±4.5%. Chamber measurements in more standard conditions, in the tumor measurements, were accurate within a more typical uncertainty of ±1.5%, accounting for standards calibration, temperature, and pressure uncertainties.

Total uncertainty, inclusive of calculation and measurement, when summated in quadrature was 2.1 and 4.7% for tissue and lung measurements respectively.

#### 2.E.2 Film measurements

As per the chamber measurements, all film measurement plans had verification cone beam CT images taken for each breath cycle to ensure correct film orientation to within ±5° and ± 1 mm. The dose attributed to the film from the CBCT acquisition was considered negligible when analysing distributions of 6 Gy fractions and was not accounted for in the comparisons. Gafchromic EBT3 film (Ashland Advanced Materials, NJ, USA), of which the accuracy has been verified in numerous works,^59^, ^60^, ^61^, ^62^, ^63^ was used for measurements. Film scanning was performed with an Expression 10000XL flatbed scanner (Epson Group, Nagano, Japan) at 24 hr post irradiation.

Dosimetric accuracy was verified using chamber measurements and 1D gamma analysis of film insert dosimetry. Gamma analysis was performed with the widely used 3%/3 mm criteria but limited to the superior inferior 1D distribution as a limitation of the phantom and possible film width. This analysis is considered acceptable for the work as the area under investigation is contained in the superior and inferior geometries, with no density changes in the remaining directional components. To ensure precision of film against the 2D dose planes, all distributions exported from the TPS were recalculated with a 2 mm dose grid for comparison. The effect of grid resolution has been shown in previous work^45^ in areas of dose build up and density junctions.

## 3 RESULTS

### 3.A Chamber measurements

The results of comparison between calculated (in the associated tumor geometry) and delivered doses to the chamber volumes are shown in Tables 1 and 2. For clarity individual reading variation is not provided, however, across all chamber readings variation per measurement were <0.3%.

**Table 1:** Percentage variation in chamber measured dose from calculated phase dose for the tumor position in each phase of the 4DCT dataset

Tumor chamber dose	Dose variation from planned					
	Inferior 2 cm	Inferior 1 cm	Plan geometry	Superior 1 cm	Superior 2 cm	Average variation
10 × 10	−1.9%	−0.7%	−0.7%	−0.8%	−2.3%	−1.3%
GTV	5.1%	1.0%	0.5%	2.2%	3.7%	2.5%
ITV	−0.5%	1.9%	0.9%	1.9%	0.8%	1.0%
Average	−3.3%	−3.2%	−3.0%	1.5%	0.8%	−1.4%
Lung override	−5.7%	−1.7%	−1.8%	0.9%	1.0%	−1.4%
Robust all	−1.2%	−0.2%	1.8%	−0.1%	−0.3%	0.0%
Robust extremes	2.4%	−1.0%	−2.4%	−0.9%	0.8%	−0.2%
Robust values	−0.5%	1.4%	0.5%	0.0%	−0.1%	0.2%
Average variation	−0.7%	−0.3%	−0.5%	0.6%	0.5%	
Standard deviation	3.3%	1.7%	1.7%	1.2%	1.7%	

**Table 2:** Percentage variation in chamber measured dose from calculated phase dose for the lung position in each phase of the 4DCT dataset

Lung chamber dose	Dose variation from planned					
	Inferior 2 cm	Inferior 1 cm	Plan geometry	Superior 1 cm	Superior 2 cm	Average variation
10 × 10	−3.2%	1.3%	1.6%	−0.2%	0.3%	0.7%
GTV	0.1%	−0.5%	3.1%	3.9%	3.1%	1.9%
ITV	0.6%	−3.2%	4.2%	2.8%	2.2%	1.3%
Average	0.7%	−2.6%	2.7%	1.6%	1.6%	0.8%
Lung override	0.6%	−0.9%	−1.2%	2.1%	2.2%	0.6%
Robust all	0.8%	2.0%	3.6%	1.8%	1.6%	1.9%
Robust extremes	1.4%	−1.2%	2.6%	1.5%	1.0%	1.1%
Robust values	0.7%	−2.9%	2.0%	2.7%	2.1%	0.9%
Average variation	0.7%	−1.0%	2.3%	2.0%	1.8%	
Standard Deviation	1.4%	1.9%	1.6%	1.2%	0.9%	

### 3.B Lung measurements

#### 3.B.1 Film Measurements

Analysis of distributions along the length of travel of the primary tumor site are presented in Table 3. Results are presented as the percentage of points passing the gamma analysis at a 3%/3 mm tolerance.

**Table 3:** 1D Gamma analysis of calculated vs film measured central axis dose profiles in plane of travel

Plan	3%/3 mm gamma result (% points passed)						
	Inferior 2 cm	Inferior 1 cm	Plan geometry	Superior 1 cm	Superior 2 cm	Average gamma	Standard deviation
10 × 10	95.8	100	98	99	98.7	98.3	1.6
GTV	93	91	99.7	100	100	96.7	4.4
ITV	100	100	91.7	96.9	98.8	97.5	3.5
Average	99.9	95.7	100	96.7	88.2	96.1	4.8
Lung override	93.7	96.7	81.4	96.7	99.1	93.52	7.0
Robust all	97.7	97.5	91.3	97	100	96.7	3.2
Robust ext	89.3	91.6	93.5	95.5	100	94.0	4.1
Robust values	97	99.9	100	95.3	98.2	98.1	2.0
Average gamma	95.8	96.6	94.5	97.1	97.9	96.4	
Standard deviation	3.7	3.6	6.4	1.6	4.0	1.8	

#### 3.B.2 Tumor dose distributions

There is strong agreement between both film and chamber dosimetry and calculations performed in each breathing phase. Given this strong agreement the planning system was utilized to quantify the dose distribution effects to the tumor and lung due to breathing motion.

**Figure 6:**
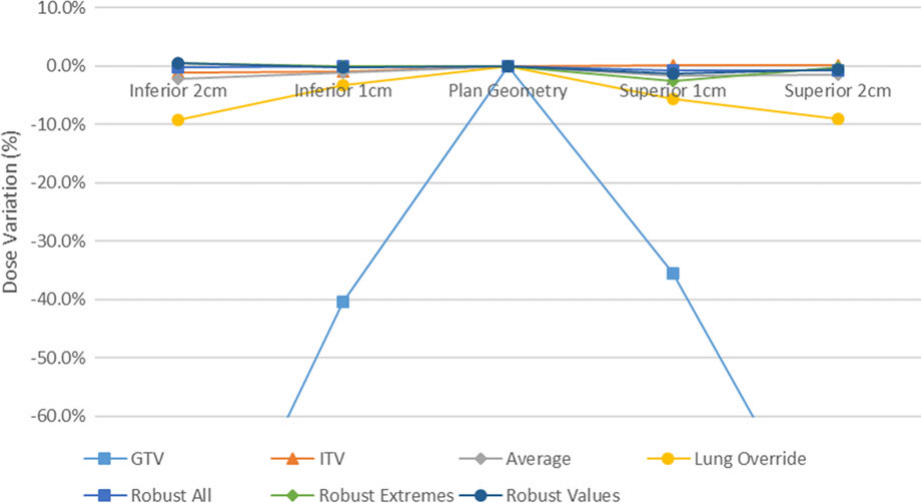
Planned 98% coverage of GTV with tumor displacement.

**Figure 7:**
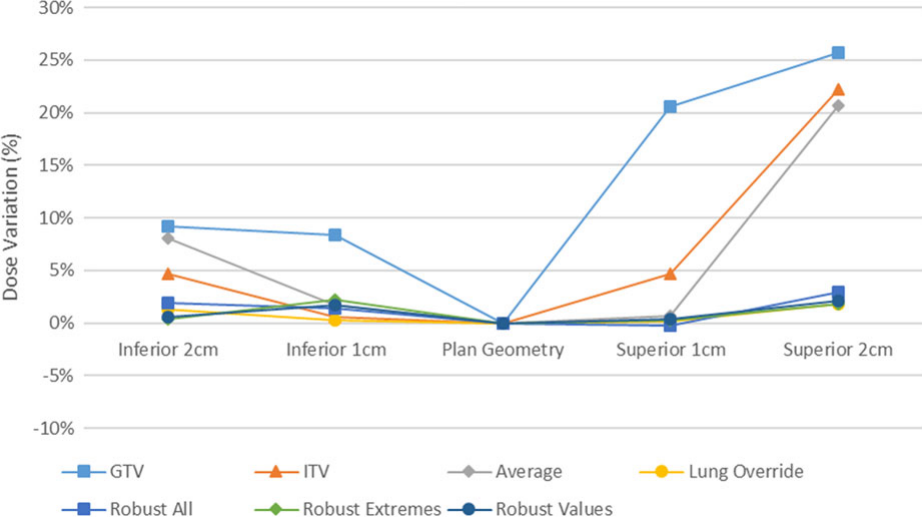
Planned GTV 2% Dose with tumor displacement.

Tumor dose variations for the various planning techniques are shown in Figs. 6 and 7. Variations from the central planned dose are displayed as a demonstration of the robustness of both coverage and resultant dose escalation from the investigated effect of tumor motion.

Adequate tumor coverage (>95%) was achieved across all breathing cycles in all plans with the exception of the GTV and Lung override plans. Both optimization techniques resulted in substantial reduction in tumor coverage with tumor motion.

Tumor dose variation was larger in the travel toward air in comparison to lung, resulting in variations in maximum dose from plan of 9, 5, 8% into lung and 26, 22, and 21% into air for GTV, ITV, and average plans respectively.

#### 3.B.3 Lung dose distributions

**Figure 8:**
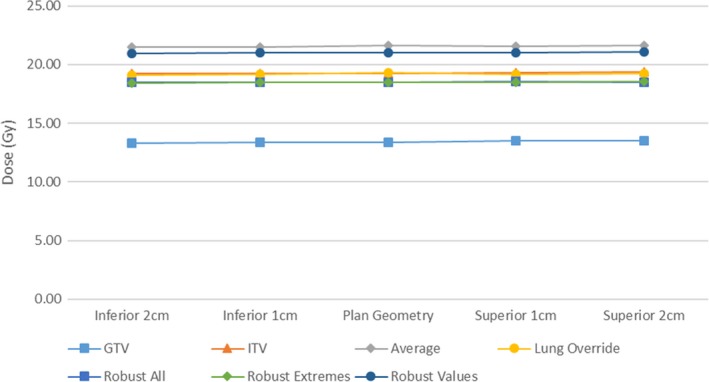
Mean Lung dose with tumor motion displacement.

**Figure 9:**
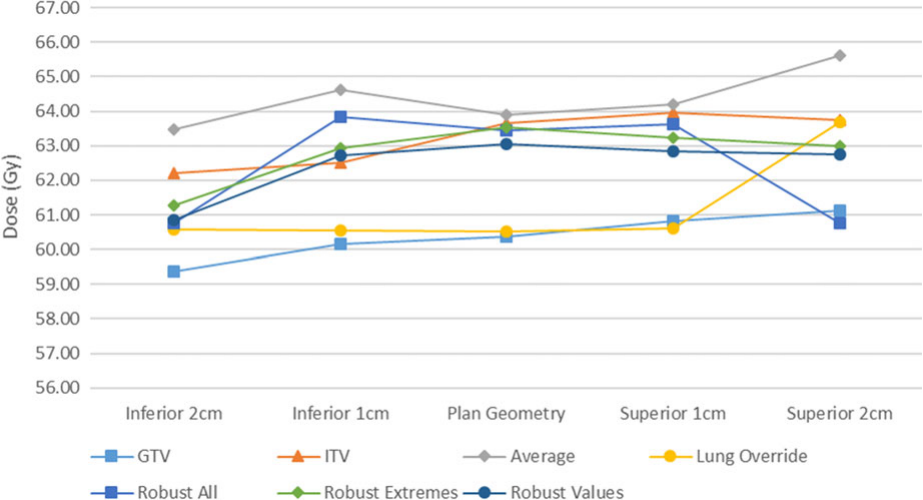
Maximum (0.03 cm^3^ volume) dose to lung (excluding GTV) with tumor motion displacement.

Measurements of lung maximum and mean dose variations are displayed in Figs. 8 and 9.

The GTV plan resulted in the lowest mean dose to the lung, a natural result of the reduced length of treatment. Of the remaining methodologies the ITV, lung override, and robust optimization across all datasets resulted in a decrease in mean lung dose of over 1.5 Gy from the average dataset methodology.

The average plan methodology produced the highest lung max dose in all breathing cycles. In all but one case the ITV plan methodology produced higher lung maximum doses than the robust optimization methodologies.

## 4 DISCUSSION

### 4.A Dose accuracy

Of the 40 central GTV measurements the mean variation from calculated dose was 0.0% ± 2.3%. The GTV plan showed the poorest agreement with an average 2.5% dose escalation from planned and a significant 5.1% dose discrepancy in the 2 cm inferior geometry.

Measurement of lung doses shows excellent agreement with calculation. Over all measurements the average discrepancy between measured and calculated dose was 1.1% ± 1.9%. All chamber measurements taken showed agreement within the uncertainty range ±6%, with a maximum discrepancy of 5.7%.

1D gamma analysis results for all 40 delivered breathing cycles are shown in Table 3. Excellent agreement is seen across all dose profiles with an average of 96.4% of all points passing the 3%/3 mm criteria, and only 2 of 40 plans resulting in a pass rate under 90%. The worst performing result was recorded with the lung override technique in the central geometry.

The results demonstrate that the TPS accurately models the delivered dose in the offset geometries for all plans. This provides the foundation for analysis of plans primarily through the Raystation planning system calculated doses.

### 4.B Plan dosimetry

**Figure 10:**
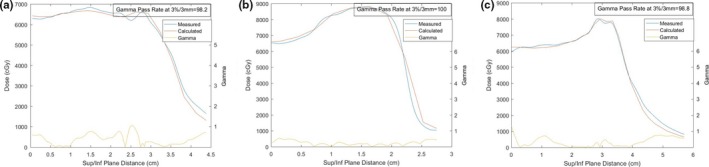
1D dose profiles and gamma analysis for 2 cm sup plans of (a) Robust All, (b) GTV and (c) ITV optimization methodologies.

**Figure 11:**
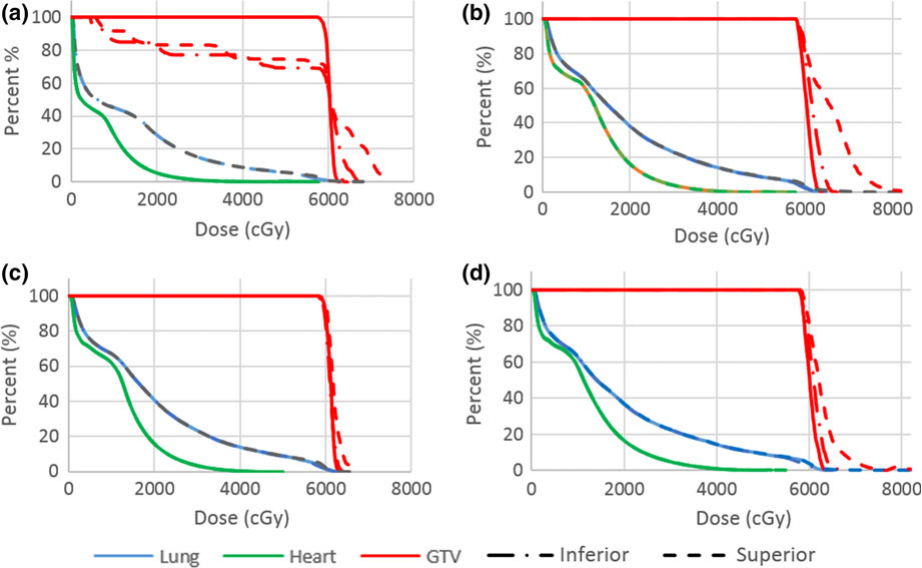
Dose Volume Histogram distributions of dose to the primary tumor, ipsilateral lung, and heart through breathing cycles 2 cm superior (air optimization) and inferior (lung optimization) for (a) GTV plans (b) ITV plans (c) Robust planning across all scans and (d) Average datasets.

The gamma analysis curves in Fig. 10 show the agreement between planned and delivered dose for the 2 cm offset toward the optimized air cavity for three different plans, verifying the effect is a real delivery consequence rather than solely computational error. It can be seen that the 130% dose escalation greater than that planned seen in the GTV and ITV plans are avoided in the robust optimization plan. This is an extreme scenario in which a 2.5 cm diameter cylinder of air resides adjacent to the tumor volume, and therefore may be clinically unrealistic. However, results in Fig. 11 show a decreased, yet similar effect with optimization to lung (Plan GTV and ITV inferior shifts). The results show parallels to previous published literature^64^ demonstrating large increases in max doses when optimizing to lung volumes.

A possible explanation for the significant dose escalation is presented by the authors. Work by Hunt et al^65^ showed reduced dose in regions adjacent to a low density inhomogeneity where the low density results in fewer electron interactions to deposit energy, greater electron path length and a potential loss of electron equilibrium. As a result, in the optimization phase additional photon fluence is required to provide equivalent dose to lung tissue and adjacent soft tissue. The effect can be paralleled to the work performed by both Thomas and Hoole^44^ and Byrne et al^45^ on the effects of optimizing to the edge of skin/surface interfaces. The presented results for internal optimization suggest that the effect is not limited to the external surface, but may also be present at depth given the existence of sufficient air or low density lung inhomogeneity. The issue occurs in phases where the GTV moves into a space optimized to air or lung tissue, where the resultant increase in electron scattering (i.e., build up and lateral scatter) due to significantly increased density results in dose escalation. When smaller targets traverse into regions that were optimized to lung density, considerable differences from the prescribed dose can occur. Such an effect is clearly represented in the DVH distributions presented in Fig. 11 for the ITV, GTV and average dataset optimized plans.

It should be noted that the effect does not appear to be linear in nature. Dose escalations when optimizing to air, 0.2 g/cm3 lung, and 0.6 g/cm3 lung were 25, 9, and 0%, respectively, compared with ratios of $\frac{\rho_{tissue}}{\rho_{air=0.001}}$, $\frac{\rho_{tissue}}{\rho_{lung=0.2}}$ and $\frac{\rho_{tissue}}{\rho_{lung=0.6}}$ of 1000, 5, and 1.66. Dose escalation is most exaggerated at edge of field boundaries. This suggests a further effect at penumbra regions where lateral electron equilibrium is lost as a result of multileaf collimator or jaw shielding.

Further work is required to determine the magnitude of dose spikes in the presence of optimization to internal air cavities and low density geometries. It is likely that the significance of such effects is impacted by a range of factors including cavity size, density of surrounding tissue, beam quality, and the presence of electron equilibrium conditions. Outside of the lung this effect may play a role in other low density regions such as sinus cavities or air in rectum and cervix patients.

In cases where large air cavities are included in the PTV within lung, such as large airways at medial lung disease, there is a clear and clinically significant dose variation. Robust optimization may be one suitable solution for such scenarios. The distributions in Figs. 6, 7, 8, 9 show significant reductions in the impact of such effects in the presence of tumor motion using robust optimization. All three robust methodologies displayed significantly less variation in max dose across the breathing cycle. Robust planning performed particularly favorably in the presence of large tumor position variations, both superiorly and inferiorly, compared with maximum variations of 25 and 8% for the GTV planning technique, 25 and 4% for the ITV technique and 23 and 5% for the average dataset planning technique.

GTV and lung override techniques resulted in insufficient dose coverage of the target over the full range of travel. Whilst this is expected for the GTV plans, this result suggests that a lung override technique is suboptimal when tumor motion is large. All other plans provided suitable coverage to the tumor across the entire motion. For the average density datasets and ITV plans this coverage was achieved at the cost of large dose variations in the doses to the GTV along its travel. In contrast, robust plans showed stable mean and max lung doses across all tumor breathing cycles. With regards to tumor dose distribution and lung doses the robust plans provided more stable dose distributions across all positional offsets, allowing for tumor dose coverage without large dose escalation.

Whether such an improvement is clinically significant is difficult to ascertain. The dose escalation of in‐air optimization for GTV, ITV and average datasets in the superior offset geometries would be considered clinically significant in almost all cases at over 20% variation. For the lung based optimization with dose escalations between 4 and 8% the blurring of dose across the breathing cycle will lead to a reduction in such a maximum. It is not the aim of this paper to produce weighted doses in line with breathing cycle such as in work by Bortfeld et al.^42^ However, given a sinusoidal breathing distribution such as outlined by Chan et al.^51^ it is not unreasonable to expect the probability of the tumor to be in the full extents of motion to around 30–40%, providing a dose variation over the breathing cycle of 3–4%. These motions also represent an extreme at a total of 4cms travel. At 1 cm superior and inferior the dose escalation results were significantly reduced, though still considerable for air optimization.

Robust optimization may have a considerable role to play in breath hold techniques given the extreme dose escalation seen in the GTV cases, even in the presence of smaller tumor motion. Further dose calculation effects may arise as a result of increased air pressure in the lungs and a reduced overall lung tissue density. The optimization across a breath hold and 1–2 maximum inhale 4DCT bins in planning may provide stable dose distributions, and reduced treatment times whilst maintaining tissue sparing.

### 4.C Further considerations

This study has intentionally focused on an extremely simple representation of the problem of optimization and tumor motion. Further to the target motion itself, the travel of surrounding tissue may have an equally large impact on the stability of the intended planned DVH distributions. Cases in which the motion of the heart traverses across several VMAT segments may have a significantly larger impact on organ and tumor dose than the effect of optimization at the tissue‐low density border and should be further investigated to assess the full potential advantages of robust optimization to clinical patients.

Implementation of robust optimization across all or partial 4DCT scans provides the fastest optimization and most theoretically pleasing solution. In this study, each GTV was provided with a 5 mm PTV expansion to account for slight changes in tumor motion and daily setup uncertainties as documented by Ruben et al.^9^ Work by McCann^66^ and Chan^51^ have shown that such uncertainties can be accounted for with reduced tissue doses by escalating dose to the edges of the GTV. Therefore, a potentially ideal solution for future investigation is the optimization across the 4DCT dataset with intentional dose escalation at the maximum extent of travel. This implementation in a current ITV approach may lead to even greater dose inhomogeneity than found in this work, and as such care should be taken.

## 5 CONCLUSION

A range of optimization techniques, including implementation of robust optimization, were used to create VMAT deliveries for moving targets in a lung phantom. All plans have been recalculated in the RayStation treatment planning system across five breathing cycles.

Chamber and film measurements were used to verify the accuracy of the RayStation calculations for each of the plans in each of the five breathing cycles. The chamber measurements show excellent agreement with the calculated dose with an average discrepancy of 0.0% ± 2.3% in the tumor and 1.1% ± 1.9% in lung. Gamma analysis performed between calculated and measured film dosimetry resulted in an average pass rate of 96.4% for 3%/3 mm criteria, with 2/40 comparisons recording pass rates under 90%.

Plans optimized using minimum dose constraints to low density volumes resulted in large dose escalations once occupied by the moving tissue density target. Dose escalations of up to 22% above planned calculation doses were noted in plans optimized to low density ITVs and average density datasets. Where possible target volumes should exclude air cavities in the lung such as bronchial airways.

Plans in which the ITV was overridden to an intermediate density resulted in reduced dose escalation but an under dose to the GTV through the breathing cycle.

Robust optimization provides greater stability in dose for large tumor motion. In the presence of smaller tumor motion of <1 cm, the effect was less significant. In areas where the PTV covered an air volume. Tumors with large motion and large density variations in surrounding tissue may result in significant improvements in dose stability with implementation of robust optimization.

## CONFLICTS OF INTEREST

The authors declare no conflict of interest.
